# Rapid histopathological imaging of skin and breast cancer surgical specimens using immersion microscopy with ultraviolet surface excitation

**DOI:** 10.1038/s41598-018-22264-2

**Published:** 2018-03-14

**Authors:** Tadayuki Yoshitake, Michael G. Giacomelli, Liza M. Quintana, Hilde Vardeh, Lucas C. Cahill, Beverly E. Faulkner-Jones, James L. Connolly, Daihung Do, James G. Fujimoto

**Affiliations:** 1 Massachusetts Institute of Technology, Department of Electrical Engineering and Computer Science and Research Laboratory of Electronics, 32 Vassar Street, Cambridge, MA 02139 USA; 2 Harvard Medical School, Department of Pathology, Beth Israel Deaconess Medical Center, 330 Brookline Avenue, Boston, MA 02215 USA; 3 Harvard-MIT Division of Health Sciences and Technology, 77 Massachusetts Ave, Cambridge, MA 02139 USA; 4 Harvard Medical School, Department of Dermatology, Beth Israel Deaconess Medical Center, 330 Brookline Avenue, Boston, MA 02215 USA

## Abstract

Rapid histopathological evaluation of fresh, unfixed human tissue using optical sectioning microscopy would have applications to intraoperative surgical margin assessment. Microscopy with ultraviolet surface excitation (MUSE) is a low-cost optical sectioning technique using ultraviolet illumination which limits fluorescence excitation to the specimen surface. In this paper, we characterize MUSE using high incident angle, water immersion illumination to improve sectioning. Propidium iodide is used as a nuclear stain and eosin yellow as a counterstain. Histologic features of specimens using MUSE, nonlinear microscopy (NLM) and conventional hematoxylin and eosin (H&E) histology were evaluated by pathologists to assess potential application in Mohs surgery for skin cancer and lumpectomy for breast cancer. MUSE images of basal cell carcinoma showed high correspondence with frozen section H&E histology, suggesting that MUSE may be applicable to Mohs surgery. However, correspondence in breast tissue between MUSE and paraffin embedded H&E histology was limited due to the thicker optical sectioning in MUSE, suggesting that further development is needed for breast surgical applications. We further demonstrate that the transverse image resolution of MUSE is limited by the optical sectioning thickness and use co-registered NLM to quantify the improvement in MUSE optical sectioning from high incident angle water immersion illumination.

## Introduction

Histopathological evaluation of thinly sectioned formalin fixed, paraffin embedded (FFPE) tissue specimens is the gold standard in many clinical scenarios including cancer diagnosis and surgical margin evaluation. Standard histological tissue processing, however, involves multiple time-consuming steps including tissue fixation, dehydration, paraffin embedding, physical sectioning and staining, and therefore typically requires at least one day before specimens can be evaluated. Frozen section analysis (FSA) is an alternative to FFPE histology that avoids lengthy fixation and paraffin embedding by freezing fresh tissue prior to physical sectioning, but still requires 15 to 30 minutes for preparation. While FSA is widely used during Mohs surgery for non-melanoma skin cancer to evaluate surgical margins, the procedure is relatively time-consuming and labor intensive, especially when multiple excisions must be evaluated. Conversely, FSA is less commonly used during breast lumpectomy surgery for breast cancer, in part because of freezing artifacts, long processing times and limited areas that can be sampled^1^. The long processing time and expense of both FFPE and FSA have motivated the development of alternative technologies that enable rapid histopathological evaluation of tissue without requiring physical sectioning.

Recently, optical sectioning microscopy has attracted interest as an alternative to FSA for rapid evaluation of pathology. Optical sectioning microscopy images a thin layer within an intact, thick specimen, greatly simplifying specimen preparation by forgoing physical sectioning. To date, various methods of histological imaging using optical sectioning microscopy have been proposed, including confocal fluorescence microscopy (CFM)^2–4^, two-photon excitation nonlinear microscopy (NLM)^5–7^, structured illumination microscopy (SIM)^8,9^, optical coherence tomography (OCT)^10–12^, and light sheet microscopy^13^. These techniques enable rapid evaluation of pathology with high correspondence to standard histological techniques^2,5^.

Unfortunately, each of these approaches has substantial limitations. NLM can image tens to hundreds of microns below the tissue surface with high resolution and strong rejection of out of focus light, but requires costly femtosecond lasers and scanning optics. CFM and light sheet microscopy avoid the use of femtosecond lasers, but still require a relatively expensive and complex optical instrument for physically rejecting out of focus light. SIM requires only inexpensive light emitting diodes (LEDs) and spatial light modulators, but rejects out of focus light computationally rather than physically, resulting in a tradeoff between available detector dynamic range and optical sectioning thickness^14,15^. OCT provides high imaging rates and deep penetration into tissue, but is incompatible with fluorescent stains and has limited ability to visualize cell nuclei which are important diagnostic features.

An alternative approach to optical sectioning, called microscopy with ultraviolet surface excitation (MUSE), was recently proposed by Levenson and Demos^16,17^. In this approach, optical sectioning is achieved by the strong absorption of deep ultraviolet (DUV) light around 280 nm wavelength by proteins, which limits fluorescent excitation primarily to the tissue surface. The optical sectioning thickness depends on the illumination angle, wavelength and tissue absorption coefficient, enabling simple imaging schemes such as ring illuminators and conventional optical microscopy without requiring DUV transmissive microscope optics, beam scanning, spatial light modulators or complex postprocessing. Furthermore, MUSE benefits from the recent commercialization of AlGaN LEDs, making inexpensive DUV light sources available at wavelengths as short as 200 nm. As a result, the cost and complexity of MUSE is very low compared to alternative imaging techniques.

To date, studies of MUSE imaging have been limited, and to our knowledge, the optical sectioning and contrast of MUSE relative to other modalities have not been explored. We have developed a MUSE system and performed a preliminary evaluation of surgical specimens for two potential applications where surgical margins are assessed intraoperatively: Mohs surgery for basal cell carcinoma of skin and lumpectomy for breast cancer. We describe a water immersion DUV illuminator using surface mount LEDs and DUV illumination guiding optics for high incident angle illumination. Increasing the illumination incident angle improves MUSE optical sectioning and contrast. We also present a staining protocol that enables rapid imaging of fresh, unfixed human tissue while minimizing the contribution of tissue autofluorescence by using a red fluorescent nuclear stain. Virtual H&E color rendering with a virtual transillumination microscopy (VTM) algorithm is used to generate images similar to standard H&E histology from two channel fluorescence detection^18^. MUSE images of human skin and breast tissue are compared with NLM images and corresponding H&E stained FFPE and FSA histology by experienced pathologists and a Mohs surgeon. The optical sectioning thickness of MUSE is quantitatively measured by using co-registered NLM volumetric images.

## Results

### Theory, design and protocol for immersion MUSE

**Figure 1:**
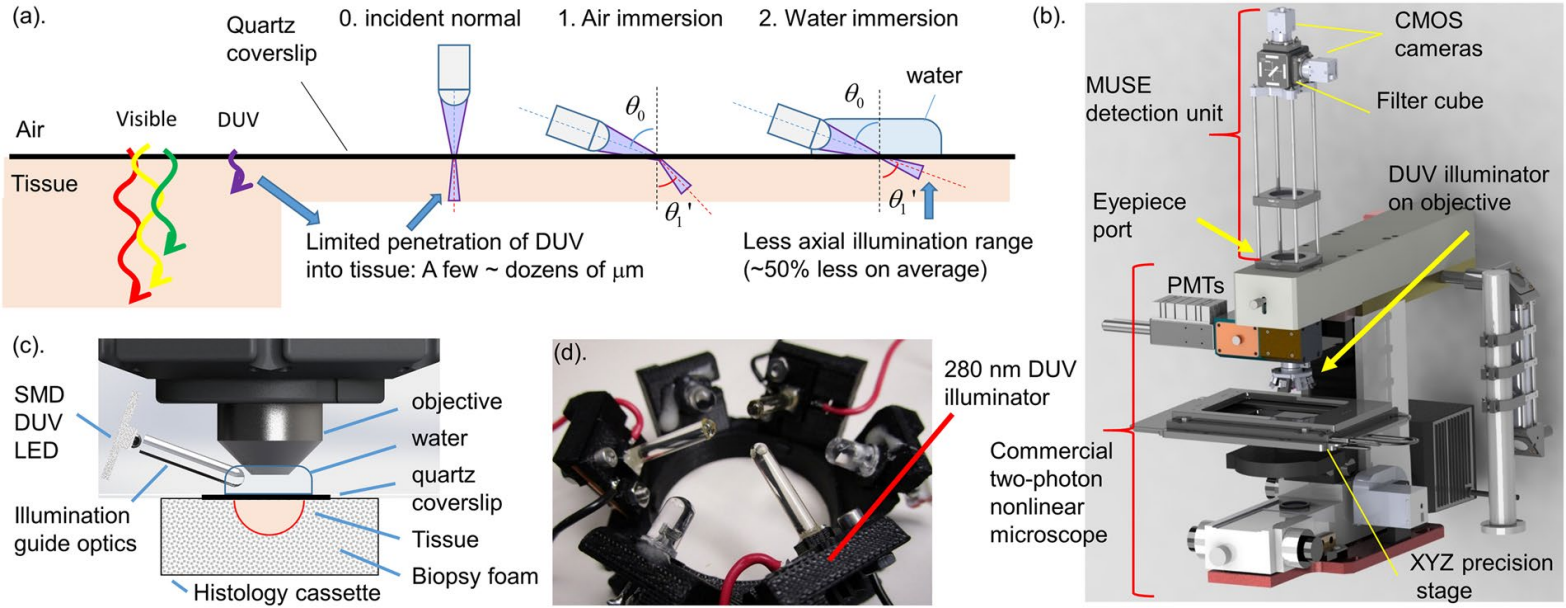
Principle of MUSE optical sectioning and MUSE imaging system. (**a**) The depth of illumination penetration into tissue decreases with wavelength and with increasing illumination angle. Higher index immersion media enables high incident angle illumination, reducing the optical sectioning thickness. (**b**) Drawing of the co-registered MUSE/NLM imaging system. MUSE imaging unit was connected to the eyepiece port of a commercial NLM system to compare MUSE images with corresponding high resolution NLM. Dual channel imaging using fluorescence filters is used for both MUSE and NLM, while a precision linear motor translation stage enables mosaic imaging. Spectrally separated fluorescence is detected by CMOS cameras (MUSE) or PMTs (NLM). DUV illuminator is mounted on the microscope objective. (**c**) Schematic of DUV illuminator with tissue specimen immobilized in a histology cassette with a quartz coverslip window and saline-soaked biopsy foam. (**d**) Photo of the illuminator with three DUV sapphire lightguides for MUSE and three visible LEDs for alignment.

MUSE achieves optical sectioning by using DUV (around 280 nm wavelength) excitation, which is strongly absorbed by proteins in human tissue, limiting fluorescence emission to the specimen surface. Although the absorption coefficient is an intrinsic tissue property, the depth of fluorescence excitation is determined by the tangential component of the illumination, which can be decreased by illuminating at a high angle, as proposed by Levenson and Demos^19^. However, the improvement in optical sectioning thickness from high angle illumination is limited by refraction at the tissue interface (Fig. 1a). Human tissue has a refractive index (n) similar to or greater than water (tissue: typical n = 1.4, water: n = 1.353 at 280 nm)^20,21^, therefore, using water immersion (or an even higher index media) will enable a steeper illumination angle relative to the surface normal (Fig. 1a). Table 1 summarizes the angle of refraction and relative axial penetration depth of DUV light for a 70-degree illumination angle. At this angle, water immersion MUSE should achieve a ~50% reduction in optical sectioning thickness for typical human tissue compared with air immersion MUSE. We designed a dual camera fluorescence imaging module which attached to the microscope eyepiece port of a commercial NLM system (Fig. 1b) and mounted 3D printed DUV illuminators on the objective (Fig. 1c,d), enabling water immersion MUSE imaging co-registered to NLM. In order to design the illuminator to support both air and water immersion, the illumination was intentionally not tightly focused, limiting acquisition speed to 8 fps or slower. Higher imaging speeds can be achieved by using separate illuminators specifically designed for air or water immersion illumination which have higher efficiencies and video rate imaging (>30 fps) should be possible using the same LED power.

**Table 1:** Relation of illumination angle and penetration depth of DUV light into human tissue (Tissue n = 1.4). The range of geometrically possible illumination angles (viewing angle to the edge of illuminator) for the system used in this paper is shown along with the central angle.

	0. normal	1. Air immersion	2. Water immersion
Angle of incidence: *θ*_0_ (degree)	0	70 (58 < *θ*_*0*_ < 83)	70 (58 < *θ*_*0*_ < 83)
Angle of refraction: *θ*_1_’ (degree)	0	42 (37 < *θ*_1_’ < 45)	63 (55 < *θ*_1_’ < 74)
Normalized axial penetration depth: *z* = cos *θ*_1_’	1	**0**.**74** (0.71 < *z* < 0.80)	**0**.**45** (0.28 < *z* < 0.57)

We developed a 2 minute specimen staining protocol using the red fluorescent nuclear stain propidium iodide (PI) and the standard histology counterstain eosin yellow (EY) dissolved in 70% ethanol or water. While PI staining of freshly excised tissue in water has been demonstrated^4^, we observed that some pathologies such as dense ducts in breast tissue specimens had limited uptake of PI. Using a 70% ethanol solvent improves PI uptake in freshly excised tissue (see Supplementary Fig. S1) and leads to more consistent nuclear staining. The microscope, imaging setup and staining protocol are described in detail under Methods.

### MUSE imaging of Mohs surgical specimens

An experienced pathologist and Mohs surgeon (BEF, DD) performed an unblinded comparison of MUSE, NLM, and FFPE H&E images of fresh, (not frozen) skin tissue discarded from Mohs surgical procedures. To compare with conventional H&E histology, virtual H&E color rendering was applied to MUSE and NLM images.

**Figure 2:**
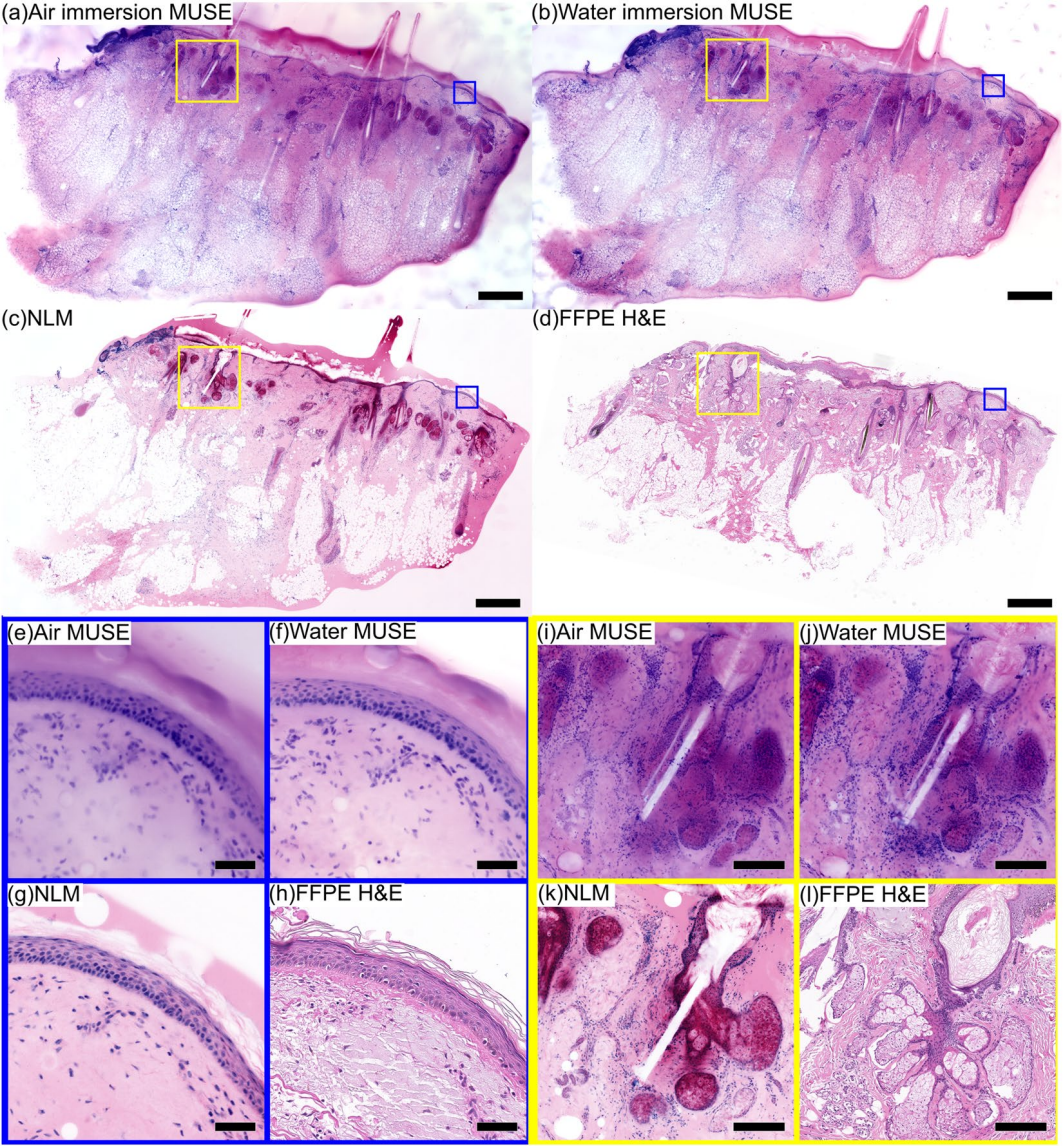
Imaging of fresh (not-frozen), normal human skin discarded during a Mohs surgical procedure. (**a**) Air immersion MUSE, (**b**) water immersion MUSE, (**c**) NLM and (**d**) FFPE H&E. The epidermis and multiple hair follicles can be seen using each modality. Magnified images of the epidermis layer (**e**) air immersion MUSE, (**f**) water immersion MUSE, (**g**) NLM, (**h**) FFPE H&E demonstrate that both air and water immersion MUSE reproduce the appearance of the normal epidermis layer, while the thinner optical sectioning of water immersion MUSE enables visualization of finer details than air immersion. Magnified images of a hair follicle and glands (**i**) air immersion MUSE, (**j**) water immersion MUSE, (**k**) NLM, (**l**) FFPE H&E demonstrate that both air and water immersion MUSE enable visualization of structures of the hair follicle (hair, sheath and glands), although some glandular structures are less clear in MUSE images because of the stacked appearance of surface and subsurface cell layers. Scale bar 1 mm (**a**–**d**), 100 µm (**e**–**h**), 200 µm (**i**–**l**). Diffuse EY can be seen both in the MUSE images and in the NLM image. However, the effect is more pronounced in the MUSE image because of the thick optical sectioning. Exposure time of MUSE: 0.3 s (water), 0.7 s (air). Air immersion MUSE: https://goo.gl/LCXYKT. Water immersion MUSE: https://goo.gl/TGfSCP. NLM: https://goo.gl/3Z66ED. FFPE H&E: https://goo.gl/UkuVfA.

Figure 2 shows a comparison of air and water immersion MUSE, NLM and conventional FFPE H&E histology of normal skin excised from the scalp of a patient undergoing Mohs surgery. PI/EY in 70% ethanol was used for MUSE and NLM fluorescent staining. The low magnification images (~60 mm^2^) show normal skin features such as epidermis, hair, follicles, sebaceous glands, and eccrine glands (Fig. 2a–d). Magnified views (Fig. 2e–h) show a clearly resolved epidermis layer for all imaging modalities, while the stratum corneum is not well visualized in both air and water immersion MUSE. At higher magnification (Fig. 2i–l), hair follicles, sheathes and sebaceous glands can be seen alongside individual hairs (white line) in all modalities. Although overall correspondence of MUSE and FFPE H&E is good, the thicker optical sectioning of MUSE compared to FFPE H&E and NLM results in some glands appearing more cellular due to the multiple cell layers at different depths visualized in MUSE images. In contrast, NLM and FFPE H&E enable differentiation between cell layers at different depths in the specimen, thereby resolving the glands without the same artifacts.

**Figure 3:**
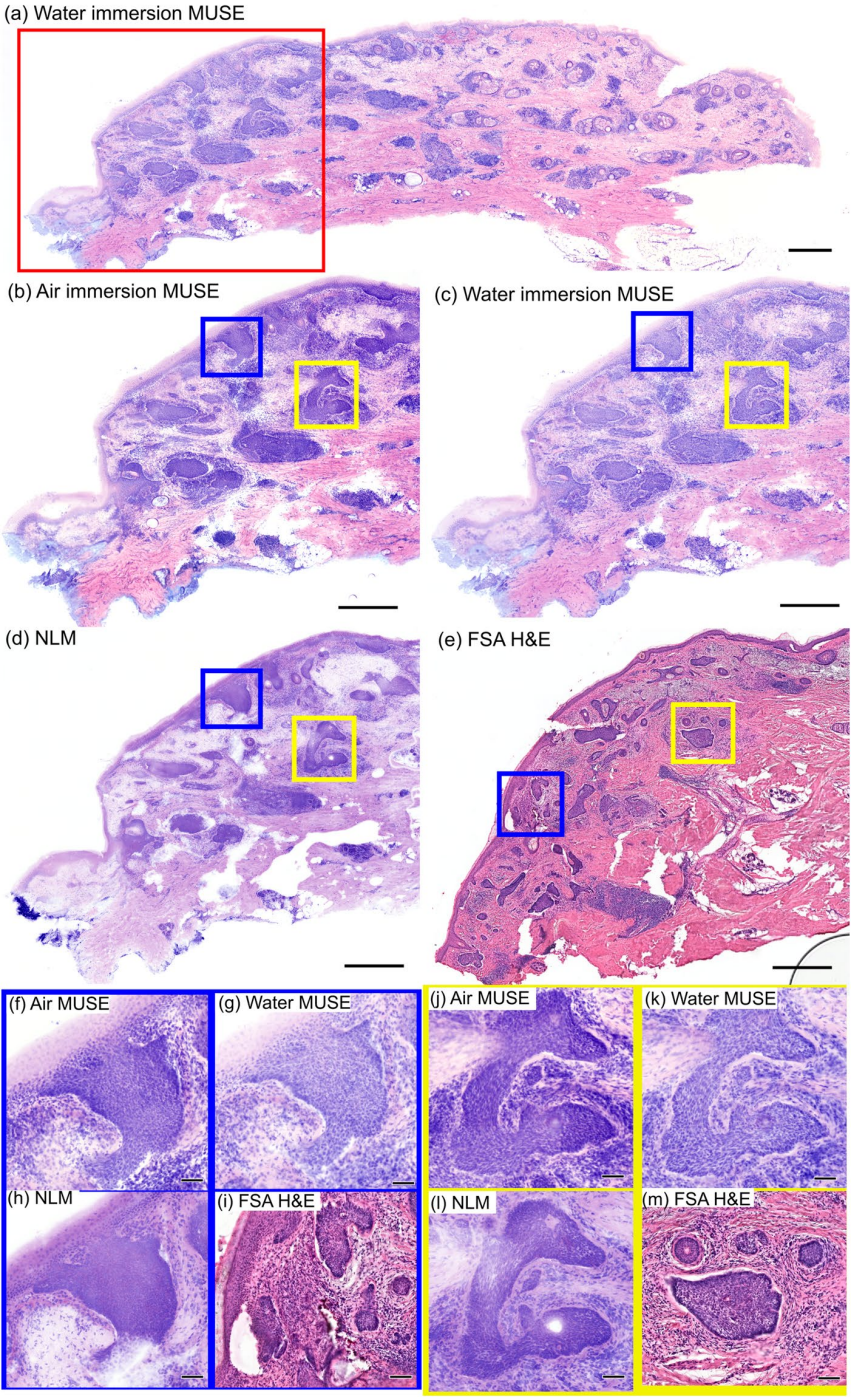
Comparison of MUSE and NLM to FSA using a 50 µm thick tissue section from the surgical margin of a discarded Mohs surgical frozen specimen. (**a**) Entire specimen imaged with water immersion MUSE. Nests of basal cell carcinoma (red box in (**a**)) visualized with (**b**) air immersion MUSE, (**c**) water immersion MUSE, (**d**) NLM and (**e**) FSA H&E. Clusters of tumor cells, reduced stroma, and normal skin structure can be visualized using each modality. Magnified images of large basaloid tumor nests with peripheral palisading (**f**,**j)** air immersion MUSE, (**g**,**k**) water immersion MUSE, (**h**,**l**) NLM, (**i**,**m)** FSA H&E suggest that both air and water immersion MUSE can reproduce the features of pathologies required for diagnosing BCC, while the thinner optical sectioning of water immersion MUSE enables visualization of detailed features better than air immersion MUSE. Scale bar 1 mm (**a**–**e**), 100 µm (**e**–**m**). Exposure time of MUSE: 0.3 s (water), 0.7 s (air). Air immersion MUSE: https://goo.gl/v4M3uw. Water immersion MUSE: https://goo.gl/4AFetK. NLM: https://goo.gl/jaET9q. FSA: https://goo.gl/JdyxHD.

To enable a direct cellular level comparison between the fluorescent imaging modalities and diagnostic FSA histology slides used during Mohs surgery, thick tissue sections (~50 µm) were taken from frozen Mohs blocks immediately above the diagnostic cutting plane. Because the thick sections are cut from the frozen specimens, this protocol enables co-registration of the FSA and MUSE/NLM fluorescent imaging planes with minimal distortion. Mosaicked images were evaluated by a pathologist and Mohs surgeon (BEF and DD). Figure 3 shows a comparison of air and water immersion MUSE, NLM and FSA H&E histology of a specimen from a patient undergoing Mohs surgery for basal cell carcinoma (BCC) located on the neck. PI/EY in water was used for MUSE and NLM fluorescent staining. The low magnification images (~50 mm^2^) show multiple large basaloid tumor nests in the papillary and reticular dermis, typical for superficial and nodular BCC (Fig. 3a–e). Magnified views (Fig. 3f–m) show basaloid tumor nests with peripheral palisading and slit-like retraction. Although water immersion MUSE achieves noticeably thinner optical sections than air immersion MUSE, features of BCC are readily identified in both cases.

### MUSE imaging of breast specimens

**Figure 4:**
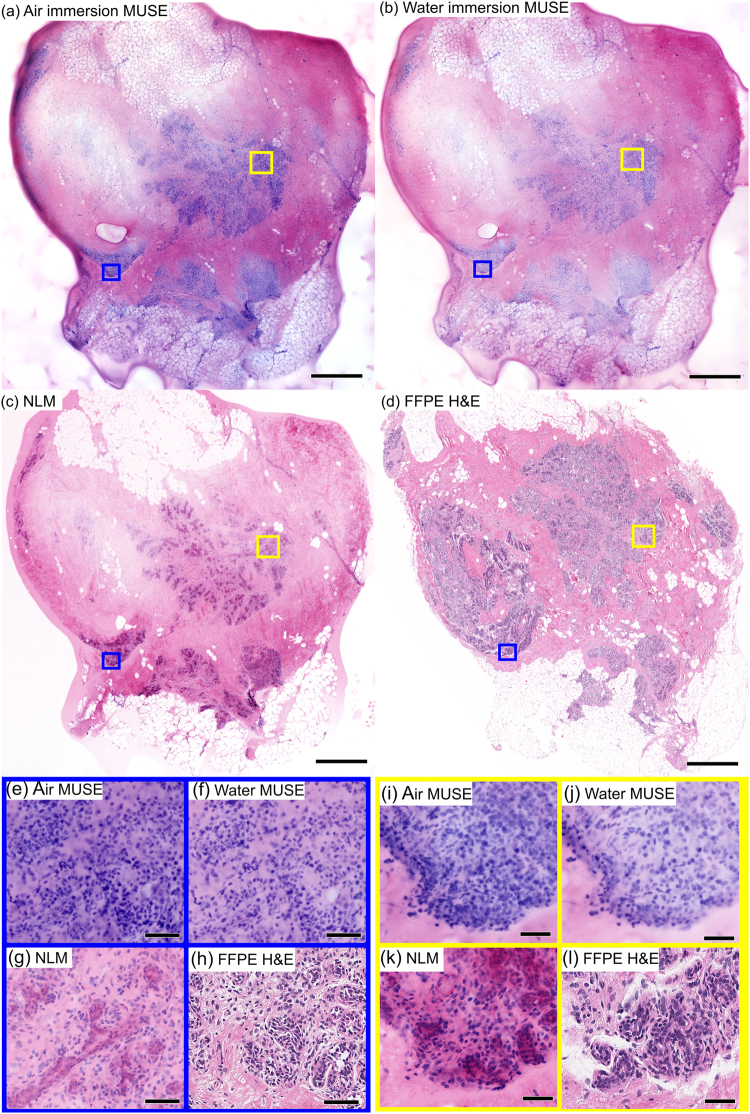
Imaging of normal breast specimen stained with PI/EY dissolved in 70% ethanol. Low magnification views of (**a**) air immersion MUSE, (**b**) water immersion MUSE, (**c**) NLM and (**d**) FFPE H&E showing TDLUs and connecting ducts. Magnified images of acini and intralobular terminal ducts with (**e**) air immersion MUSE, (**f**) water immersion MUSE, (**g**) NLM and (**h**) FFPE H&E, and another lobule with (**i**) air immersion MUSE, (**j**) water immersion MUSE, (**k**) NLM and (**l**) FFPE H&E. The thicker optical sectioning in MUSE limits accurate visualization of lobular structures. Scale bar: 1 mm (**a**–**d**), 100 µm (**e**–**h**), 50 µm (**i**–**l**). Exposure times of MUSE: 0.125 s (water), 0.300 s (air). Air immersion MUSE: https://goo.gl/xoeiS4. Water immersion MUSE: https://goo.gl/jWVWnD. NLM: https://goo.gl/TdSnxF. FFPE H&E: https://goo.gl/NvAhd3.

Three experienced pathologists (LQ, HV, JC) performed an unblinded qualitative comparison of air and water immersion MUSE, NLM and FFPE H&E histology of fresh, unfixed normal human breast tissue imaged shortly after mastectomy (Fig. 4). PI/EY in 70% ethanol was used for MUSE and NLM fluorescent staining. The low magnification images of normal breast tissue (~25 mm^2^) show terminal ductal lobular units (TDLUs), normal adipocytes and stroma (Fig. 4a–d). Magnified images of TDLUs, including the acini and intra-lobular terminal ducts, show high correspondence between NLM and FFPE H&E (Fig. 4g,h); however, visualization of the lobular structure is limited in MUSE images (Fig. 4e,f). Magnified images of another lobule clearly shows the lobular structure on NLM and FFPE H&E (Fig. 4k,l), but MUSE images (Fig. 4i,j) do not resolve detailed acinar structures, although the boundary of the lobule is apparent. Water immersion MUSE images show fewer artifacts than air immersion MUSE, but the optical sectioning is still insufficient and evaluation is difficult. All three pathologists agreed that the general architecture of lobules are not identifiable in high magnification MUSE images because of a lack of stromal contrast and an appearance of overly dense nuclei due to the thicker optical sectioning.

### Surface contamination in optical sectioning microscopy

**Figure 5:**
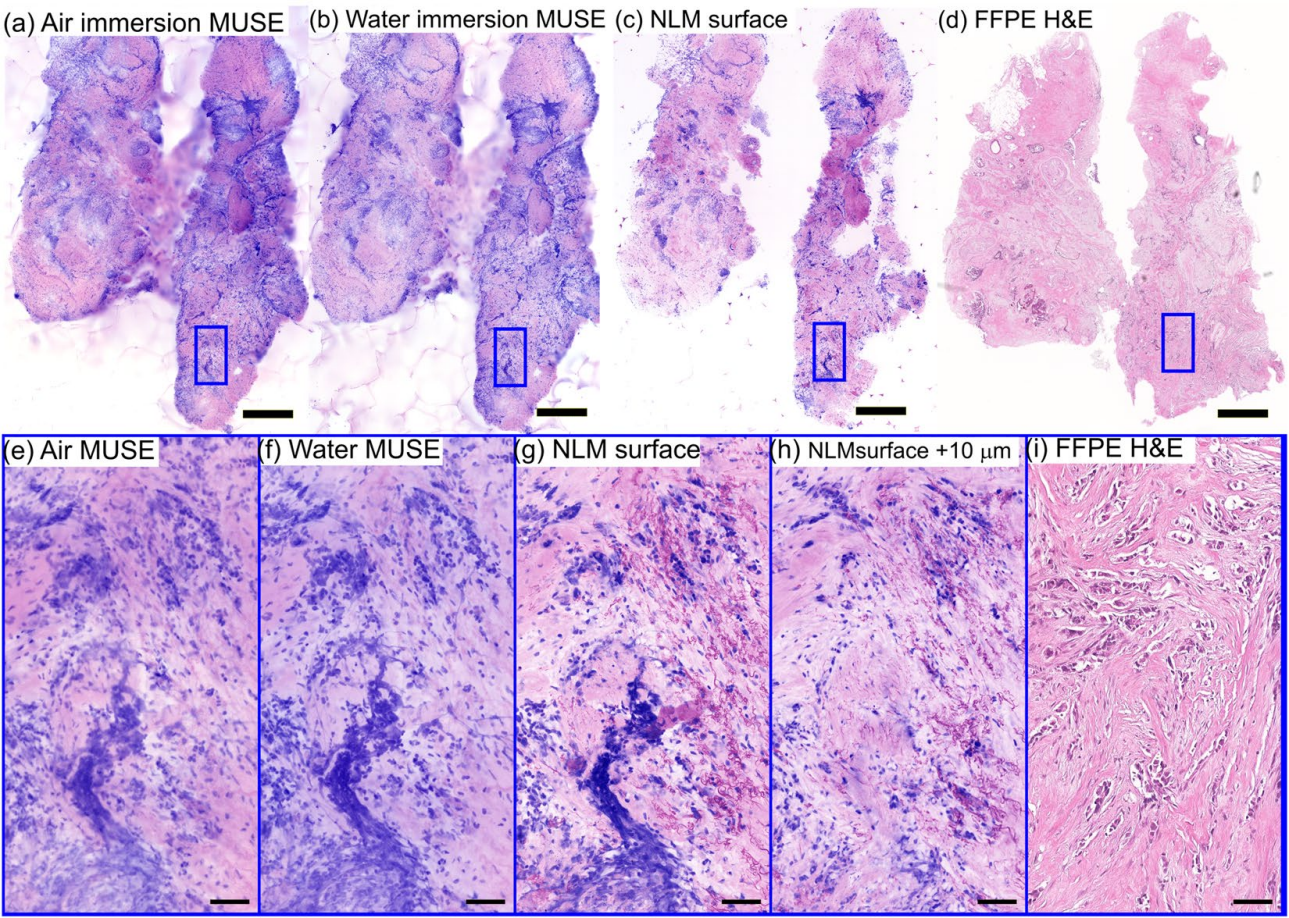
Imaging of a breast specimen with high grade invasive ductal carcinoma stained with PI/EY dissolved in water. (**a**) Air immersion MUSE, (**b**) water immersion MUSE, (**c**) NLM and (**d**) FFPE H&E images show a high density of cells from high grade invasive ductal carcinoma and surface contamination. Magnified views of (**e**) air immersion MUSE, (**f**) water immersion MUSE, (**g**) NLM at the specimen surface shows aggregated cells displaced from original sites which can be difficult to distinguish from invading cancer cells. Magnified view of (**h**) NLM at 10 µm below surface shows the tissue structure without surface contamination artifacts. (**i**) Magnified view of FFPE H&E shows invasive cancer without surface contamination artifacts. Surface contamination is not present in conventional histology because it is removed during microtoming. Scale bar: 1 mm (**a**–**d**), 100 µm (**e**–**i**). Air immersion MUSE: https://goo.gl/7mW2MR. Water immersion MUSE: https://goo.gl/4ZJMLC. NLM: https://goo.gl/xsAzKv. FFPE H&E: https://goo.gl/jECgfa.

MUSE has potential limitations for intraoperative applications because it images the tissue surface. Cauterization or cellular debris may contaminate the tissue surface during surgery or grossing and can interfere with evaluation. Conventional FFPE histology and FSA do not have surface contamination artifacts because the specimen surface is removed by facing the embedded tissue block during microtoming. Figure 5 shows mosaicked images with air and water immersion MUSE, NLM and FFPE H&E histology of a breast specimen with high grade invasive ductal carcinoma (IDC) (~20 mm^2^). PI/EY in water was used for MUSE and NLM fluorescent staining. Loose tumor cells and other debris from grossing are present in the MUSE and NLM images of the tissue surface (Fig. 5a–c) and interfere with image evaluation. Magnified images of the tissue surface with both MUSE and NLM (Fig. 5e–g) show contamination by aggregated cells that obscure the underlying tissue and may be mistaken for invading tumor cells. Subsurface imaging with NLM (Fig. 5h, 10 µm below the surface) demonstrates that the aggregated cells are not present in the underlying tissue. This is confirmed by FFPE H&E histology which has a histological section plane deeper in the tissue, below the NLM image planes (Fig. 5d,i).

### Relationship between MUSE optical sectioning and image resolution

**Figure 6:**
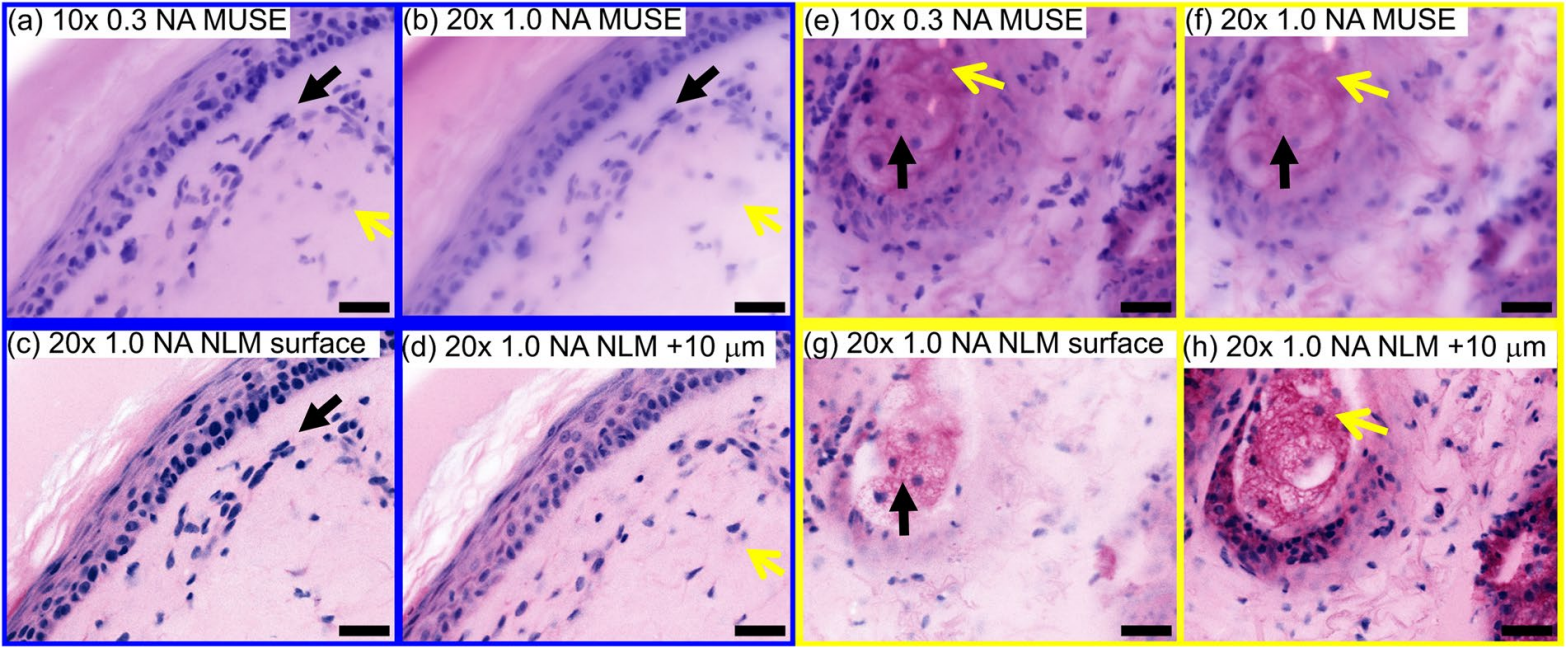
Relationship between MUSE optical sectioning thickness and transverse image resolution/NA of the objective. Comparison of (**a**,**e**) 10x, 0.3 NA water immersion and (**b**,**f**) 20x, 1.0 NA water immersion MUSE with (**c**,**g**) NLM image of tissue surface, and (**d**,**h**) 10 µm below the surface. MUSE at 20x, 1.0 NA shows poorer transverse resolution compared with 10x, 0.3 NA because the MUSE optical sectioning thickness is an order of magnitude larger than depth of field of the 20x, 1.0 NA objective. Fluorescence from depths which are out of focus blurs the image. Black arrows indicate nuclei on the tissue surface and yellow arrows indicate nuclei 10 µm below the surface. Scale bar 50 µm.

Unlike NLM or CFM, MUSE optical sectioning is independent of the imaging numerical aperture (NA). Therefore the NA and magnification can be changed while maintaining a constant optical sectioning thickness. However, the maximum NA and transverse image resolution is limited because the optical sectioning thickness should not exceed the image depth of field. To demonstrate the effect of NA and depth of field on image resolution, water immersion MUSE images of fresh, unfixed normal skin were acquired with a 20x, 1.0 NA objective, a 10x, 0.3 NA objective, and compared with NLM using a 20x, 1.0 NA objective (Fig. 6). The 1.0 NA objective has an order of magnitude less depth of field (1.5 µm) than the optical sectioning thickness of water immersion MUSE (10 µm, see following section), while the 0.3 NA objective has a depth of field (10 µm) comparable to the MUSE optical sectioning thickness. NLM images at (Fig. 6c,g) and 10 µm below the surface (Fig. 6d,h) show that both MUSE images (10x, 0.3 NA MUSE: a, e, 20x, 1.0 NA MUSE: b, f) enable visualization of nuclei at (black arrow) and below the tissue surface (yellow arrow). However, the higher, 1.0 NA MUSE images exhibit decreased transverse resolution because fluorescence from depths which are out of focus contributes to the image and causes blurring. In contrast, the 0.3 NA MUSE image has a longer depth of field, maintaining focus over most of the depth range of the fluorescence. Using higher index immersion media should extend the usable NA for MUSE because it will increase depth of field while further decreasing optical sectioning thickness.

### Quantitative assessment of optical sectioning thickness in MUSE

Measuring the optical sectioning thickness in MUSE is challenging because the absorption depth of the DUV illumination depends on the tissue properties and can also have local variation. Furthermore, in contrast to NLM or CFM, acquiring images at multiple depths (z-stacks) in MUSE only changes the focal plane depth without changing the optical sectioning from DUV absorption, precluding the use of traditional microscopy characterization methods. To determine the optical sectioning thickness of MUSE in human tissue, we developed a method that images individual cell nuclei sparsely located at different depths. In this method, the depths of nuclei are measured precisely using volumetric NLM images acquired at 1.0 NA, and then the same tissue specimens are imaged using MUSE. By correlating the signal intensity measured with MUSE with the depth information obtained from volumetric NLM, the reduction in MUSE signal as a function of depth below the tissue surface can be computed. Furthermore, by measuring an ensemble of nuclei, random variations in local tissue absorption or nuclei brightness can be averaged to determine an effective overall measure of MUSE optical sectioning.

**Figure 7:**
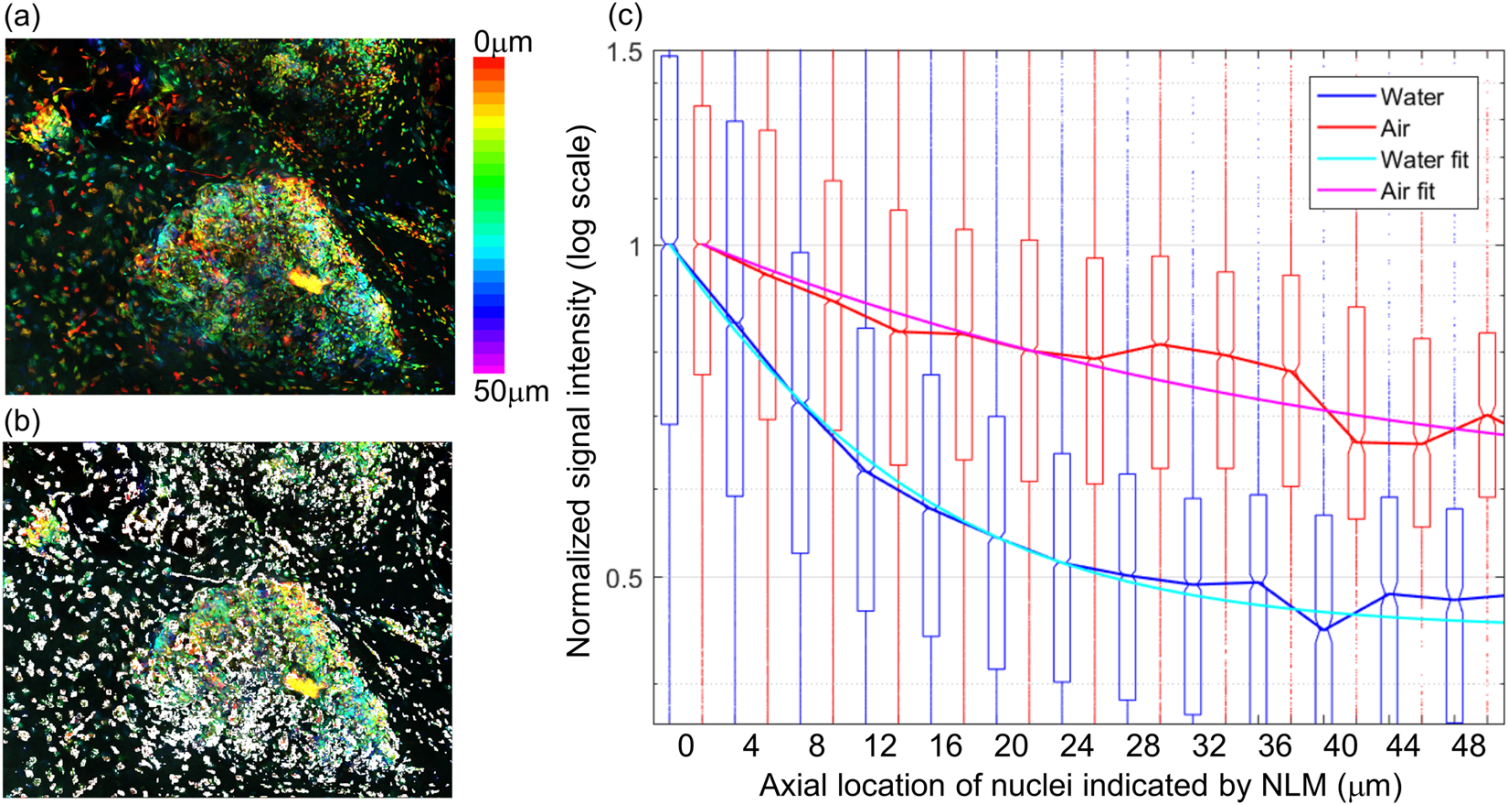
Quantitative assessment of optical sectioning thickness in MUSE. (**a**) Volumetric NLM images of fresh, unfixed benign breast tissue (~1 mm^2^) were used to measure the depth of nuclei observed in MUSE. Tissue was stained with PI/EY in water; but only the PI channel signal was used for this analysis. Isolated nuclei were segmented ((**b**), white areas. Total 0.3 mm^2^) and the signal intensity of nuclei in co-registered water immersion and air immersion MUSE images were calculated as a function of depth measured by NLM. (**c**) Box plot of the signal intensities for each depth shows that water immersion MUSE suppresses signal from nuclei deep inside tissue substantially more than air immersion MUSE. Boxes shows 25–75% range, solid lines show median, and small dots show outliers. Fitting curves indicate that the *e*^−1^ depths are 10 µm (water immersion MUSE) and 20 µm (air immersion MUSE).

This method was applied to fresh, unfixed benign human breast tissue (Fig. 7a). Volumetric NLM images were co-registered with air and water immersion MUSE images from the specimen surface. The NLM and MUSE images were divided into ~260,000 areas (4 µm^2^), of which ~77,000 areas containing isolated nuclei were used for analysis (Fig. 7b, white area). Areas without nuclei or with nuclei at multiple depths were excluded. Each area was then sorted by depth as measured by NLM, and the MUSE intensity for all areas at each depth were grouped for analysis. Figure 7c shows a bar plot of the median and range (25–75 percentile) of signal intensities from nuclei for air and water immersion MUSE as a function of depth. The solid line fits were calculated by modeling the nuclei fluorescence as a combination of a DUV excitation term following Beer’s law and a constant background fluorescence term; $(1-a)\exp (-d/{d}_{0})+a$ ($a$: constant background fluorescence, $d$: depth of nuclei, ${d}_{0}$: *e*^−1^ depth). The average calculated *e*^−1^ depths were 10 µm and 20 µm for water and air immersion MUSE respectively. Although there was considerable variation in fluorescence intensity and attenuation with position, as evident by the large 25–75 percentile range, water immersion improved MUSE optical sectioning at all depths below the surface. The calculated ratio of the DUV excitation term to the constant background term was 1.22 for water immersion MUSE and 0.56 for air immersion MUSE.

## Discussion

MUSE is a promising method for rapid histological evaluation of surgical specimens at lower cost than other optical sectioning microscopy technologies such as NLM or CFM. We designed and characterized air and water immersion MUSE systems using high incident angle illumination delivered with illumination light guiding optics. We developed a tissue staining protocol using PI as a fluorescent nuclear stain and EY as a counterstain. In contrast to other nuclear stains such as acridine orange (AO) which have been used to stain skin and breast tissue^2,5^, PI is particularly advantageous for MUSE because the red fluorescence emission is at longer wavelength than the dominant tissue autofluorescence. Images were acquired using low-cost machine vision cameras. We demonstrated that MUSE can generate virtual H&E images of fresh, unfixed human tissue using a rapid sample staining protocol.

We performed a preliminary investigation to assess the feasibility of MUSE for surgical pathology of skin and breast cancer. Based on an unblinded, qualitative assessment of MUSE, NLM and FFPE or FSA H&E histology by an experienced pathologist and Mohs surgeon, MUSE images reproduced many of the diagnostic features of human skin specimens seen on FFPE and FSA H&E. Features of BCC pathology such as large nests of basaloid tumor cells, clefts, and atypical appearance of palisading nuclei were apparent on MUSE images, as were features of normal skin such as the epidermis and glands. Although there was general consensus that the contrast of nuclei and stroma in MUSE images was less than that of FFPE and FSA H&E, the architectural features of BCC could be visualized in MUSE. These results suggests that MUSE may be a promising method for evaluating skin pathology more rapidly than FSA, potentially reducing processing time from 15–30 minutes to a few minutes.

In contrast, experienced breast pathologists had difficulty in interpreting MUSE images of normal breast tissue when compared to FFPE H&E or NLM. The thicker optical sectioning of both air and water immersion MUSE leads to the appearance of acini (~50 µm diameter) as relatively homogenous structures, instead of segmented glands with open lumens typically seen on FFPE H&E and NLM (Fig. 4). Furthermore, MUSE images of breast tissue had low contrast in the EY channel that made interpretation of some dense stromal features challenging. Pathologists also had differing opinions on the extent to which the increased cellularity present in the thicker MUSE optical sections affected low magnification interpretation of structures, suggesting that more extensive experience in interpreting MUSE images or improvements in resolution and contrast may be required for breast pathology applications.

Artifacts from surface contamination may pose limitations for intraoperative applications using MUSE. Unfixed breast tissue specimens exhibited surface contamination from grossing and handling procedures, such as displacement of cells from other areas, smeared or transferred tissue marking inks, and other debris, creating a possible false appearance of pathology and obscuring underlying tissue features. These contamination artifacts can be reduced with careful handling, but may still be present to some degree. Surface contamination can be avoided in NLM by imaging 10~20 µm or more below the tissue surface. Controlling surface contamination appears to be much easier for smaller Mohs specimens than for larger breast tumors, for scalpel rather than for electrocautery excisions, and for solid rather than for friable specimens.

As expected, high angle illumination with water immersion MUSE improves optical sectioning thickness by a factor of two, while reducing background fluorescence. This was qualitatively assessed using mosaic images and quantitatively confirmed using co-registered volumetric NLM to measure depth of cell nuclei contributing to MUSE images. Quantitative measurements with fresh, unfixed benign breast tissue indicate that water immersion MUSE has a 10 µm e^−1^ optical sectioning thickness, while air immersion MUSE has a 20 µm e^−1^ thickness, although this does not include the contribution from the background fluorescent signal and absolute thicknesses vary within individual specimens. This difference in optical sectioning thickness is consistent with the predicted improvement based on simple geometrical optics arguments (Table 1). Unfortunately, we could not quantitatively measure MUSE optical sectioning thickness in skin specimens due to the high density of nuclei and non-specific PI staining of skin stroma, but subjectively the optical sectioning thicknesses appeared similar in skin and breast.

The presence of a diffuse background fluorescent signal that is independent of tissue surface features (Fig. 7c) suggests that an additional mechanism excites fluorescence below the surface. Scattering may contribute to the background fluorescence because the scattering mean free path at 280 nm wavelength in many human tissues is estimated to be 30~60 µm^22,23^, which is only modestly larger than the absorption mean free path of 20~25 µm, calculated from the measured optical sectioning thickness and illumination angle. Another possible mechanism is secondary excitation of exogenous fluorophores (e.g. EY/PI) by tissue near-UV autofluorescence. The optical sectioning of superficial tissue layers is presumed to be governed by the strong attenuation at 280 nm wavelength by amino acids, primarily tyrosine and tryptophan. However, both tyrosine and tryptophan also fluoresce with quantum yields of 0.12 and 0.13 and emission maxima of 300 nm and 350 nm respectively^24^. The relatively large stokes shift of the tryptophan emission may cause secondary excitation of deep underlying tissue layers. Likewise, secondary excitation by EY fluorescence may also contribute to a background signal in the PI channel. Finally, we note that primary tissue autofluorescence does not directly contribute to the background because the choice of red-shifted fluorophores and emission filters reduced the autofluorescence of unstained tissue to negligible levels. Further measurements and modeling are required to understand the extent to which scattering and secondary near-UV fluorescence influences optical sectioning and contrast in MUSE, however, the use of fluorophores such as PI that have relatively low absorption in the near-UV may improve performance compared with near-UV excitable agents such as DAPI or Hoechst 33342.

The ultimate resolution and contrast limits of MUSE remain an open question. While MUSE can be performed at low to moderate NA, the requirement that the optical sectioning thickness approximately matches the depth of field means that higher NA imaging will not improve transverse image resolution unless the optical sectioning thickness can be reduced. In fact, our results demonstrate that 0.3 NA is well-matched to the optical sectioning thickness of the water immersion MUSE system, while 1.0 NA has degraded image resolution because the optical sectioning thickness is larger than the depth of focus. Although we have shown that water immersion can markedly improve both optical sectioning and contrast, it is likely that further improvements are possible using even higher index immersion, narrower ranges of illumination angles (e.g. from spatially coherent light sources), and staining protocols that minimize contributions from Stokes-shifted secondary autofluorescent emission.

Further experimental and modeling studies are required to understand how MUSE imaging hardware and staining protocols can be optimized. The preliminary findings in this study suggest that current air and water immersion MUSE may not be suitable for intraoperative breast cancer applications because of limitations in optical sectioning thickness and ability to image below surface contamination. At the same time, findings suggest that MUSE may be promising for BCC in Mohs surgery because this application exhibited much greater correspondence between MUSE and H&E histology, and because specimens exhibited less surface contamination. Larger scale studies of skin pathology comparing blinded reading of MUSE to conventional H&E histology are warranted to assess sensitivity and specificity.

## Methods

### MUSE illuminator

Efficient illumination of the tissue surface with DUV LEDs is challenging because LED modules typically do not fit into the short working distance of an immersion objective. To effectively couple DUV light to the field under the microscope objective, we developed a novel light guiding illuminator using surface mount, high power 280 ± 5 nm wavelength LEDs (Nikkiso, VPS161), sapphire rods (3 mm dia.), and quartz and sapphire hemisphere lenses (3 mm and 2.5 mm dia. respectively). The high refractive index of sapphire produces total internal reflection of the wide angle DUV light emission from the surface mount LED (130 degree viewing angle), efficiently guiding light to the region of interest. The use of inexpensive sapphire rods, quartz and sapphire hemisphere lenses reduces system cost, while minimizing the amount of immersion fluid required, enabling surface tension to draw immersion media onto the illuminator and under the objective.

The power from a single LED was 25 mW measured at the LED surface and 6 mW from the lightguide, a 24% efficiency. Losses include: reflection loss between air, rod and lenses (~40%) and incomplete total internal reflection, mainly between the sapphire rods and diodes, due to the very large divergence angle of the diodes. Reflection loss could be reduced by index matching interfaces and incomplete total internal reflection could be reduced with additional coupling optics. Three DUV LEDs and lightguide illuminators spaced 120 degrees apart to avoid shadowing were assembled on a 3D printed holder. Using water immersion, the illumination was quasi-collimated with a beam width of ~4 mm, and the total illumination area was ~12 mm^2^. Using air immersion, the refraction at the hemispherical output lens focused the illumination before the coverslip which then diverged to an illumination area of 50 mm^2^ at the coverslip. For the illuminator to function with both water and air immersion objectives, a wide area relative to the field of view was illuminated, resulting in a low fluorescence signal and low imaging frame rate. Video-rate imaging (>30 fps) should be possible if different illuminator designs are used for air vs water immersion.

### Imaging system

The MUSE imaging system was designed to be integrated into a commercial NLM system (Thorlabs Inc.) using the eyepiece port. The fluorophores PI and EY were selected as nuclear- and counterstain because of their relatively long wavelength emission, which minimizes the contribution of the short wavelength autofluorescence and secondary emission from the LEDs. Therefore, no excitation filter was required. Fluorescent light from the tissue was separated by a dichroic beam splitter (cutoff of 590 nm) into two spectral channels and additional band-pass filters from 520 to 560 nm and from 620 to 680 nm were used for EY and PI detection, respectively. Images were acquired with two low-cost monochrome CMOS cameras (Point Grey BFLY-U3-23S6M: 41 fps at 1920 × 1200 resolution and 12 bits per pixel) in the MUSE system or by two photomultiplier tubes (PMTs) (H7422; Hamamatsu, Inc.) in the NLM system. Two 10x, 0.3 NA objectives (air immersion: Olympus MPLFLN10X, water immersion: Zeiss Achroplan 10x/0.3 W) were used to compare air and water immersion MUSE at equal NA. A 20x, 1.0 NA water immersion objective (Olympus XLUMPFL20XW) was also used for NLM as well as to investigate high NA MUSE imaging (Fig. 6). The depths of field of the objectives were calculated to be 7.5 µm (10x air), 10 µm (10x water) and 1.5 µm (20x water), respectively. The fields of view of the MUSE system were 0.68 × 0.47 mm (10x Air), 0.62 × 0.43 mm (10x Water), 0.35 × 0.24 mm (20x Water), with an effective 1680 × 1150 pixel image after digital alignment of the two spectral channels. MUSE image acquisition was performed with 125 to 300 ms exposure time (up to 8 frames per second equivalent) for water immersion MUSE and 500 ms to 1 s exposure time for air immersion MUSE using unity detector gain (1 count per photoelectron). The long exposure time for air immersion MUSE was due to the wider illumination area produced when the illuminator was used in air. Air immersion MUSE with the same illumination area as water immersion MUSE would achieve similar imaging speed. The imaging speed also depends on the detector gain and could be increased for preview or live imaging at the expense of a minor decrease in dynamic range, which far exceeds the signal to background ratios observed in MUSE images. A ∼150-fs pulse duration tunable Ti:sapphire femtosecond laser (Mira Optima 900-F; Coherent) with a 76-MHz repetition rate operating at ~780 nm wavelength was used for NLM imaging. A detachable primary dichroic mirror was used to direct specimen fluorescence to the MUSE cameras and the NLM PMTs. For NLM, the femtosecond laser was scanned using a nonresonant galvanometer scanner (slow axis) and an 8 kHz (16 kHz bidirectional) resonant galvanometer scanner (fast axis). The NLM operated at 16 fps with <30 mW average power incident on the specimen. Each NLM image frame was acquired at 1024 × 1024 pixels, with a 0.49 mm field of view.

### Specimen preparation

Freshly excised skin tissue, thick frozen sections (~50 µm) of skin tissue, stored in ethanol following standard clinical practice, digital images of frozen section slides, and freshly excised breast tissue were acquired under protocols approved by the Massachusetts Institute of Technology Committee on the Use of Humans as Experimental Subjects (COUHES) and the Beth Israel Deaconess Medical Center (BIDMC) Committee on Clinical Investigations (CCI). Only discarded and de-identified human tissue specimens that were no longer required for clinical diagnosis were used, and the protocol was therefore exempt from informed consent. All experiments were performed in accordance with the protocols. The discarded, freshly excised skin and breast tissue specimens were maintained in chilled Roswell Park Memorial Institute (RPMI) medium. All freshly excised tissues were imaged within 12 hours of excision. The fresh skin and breast specimens were transected to expose representative regions (3 to 5 mm width) of stroma or pathology immediately prior to staining and imaging to minimize surface contamination. The freshly transected skin and breast tissue specimens were stained for 2 minutes in a solution of 40 μg/ml PI and 200 μg/ml EY dissolved in 70% ethanol or in water as noted in each experiment. We observed that using a 70% ethanol solvent improves uptake of PI into fresh tissue, especially in dense ducts and lobule of breast as compared to using a water solvent (Supplementary Fig. S1). Except for uptake of PI, there was no change observed in the specificity and fluorescence intensity for both PI and EY staining with 70% ethanol vs water. Therefore PI/EY in 70% ethanol staining protocol was used for the qualitative comparison of images from fresh, unfixed tissue (Figs 2 and 4). The stained fresh, unfixed specimens were rinsed in buffered saline and placed on a 200 µm thick quartz glass coverslip attached to a histology cassette. The histology cassette was filled with saline-soaked biopsy foam pads so that the specimen surface was immobilized and compressed against the quartz coverslip without dehydration. This method enables repeated imaging of the same specimen for several hours without noticeable alteration of cellular structure or fluorescent signals. Thick tissue sections taken from frozen Mohs blocks were maintained in ethanol and stained for 2 minutes in a solution of 40 μg/ml PI and 200 μg/ml EY dissolved in water. The stained thick tissue sections were then rinsed in buffered saline and covered by a 200 µm thick quartz glass coverslip. Specimens were rehydrated in water during staining/rinsing because ethanol rapidly evaporates during imaging.

### Imaging procedure

Large tissue areas (~50 mm^2^) were imaged consecutively with MUSE and NLM using a high precision linear motor stage (MLS-203, Thorlabs, Inc.). NLM images at multiple depths (z-stacks from surface to ~50 µm depth) were also acquired to quantitatively assess the optical sectioning thickness of MUSE. During MUSE and NLM imaging, tissue specimens were kept immobilized in the histology cassette. After MUSE and NLM imaging, the skin and breast tissues were fixed in 10% formalin for more than two days, and processed for conventional FFPE H&E histology. Since the digitally scanned images of actual diagnostic frozen sections used to guide surgery were available for Mohs specimens, these images were used for comparison of the skin cancer specimens. A team of experienced pathologists and a Mohs surgeon (LQ, HV, BEF, JC, DD) evaluated the mosaicked images of each modality at full-resolution. No image processing was performed for the images used in the quantitative analysis (Fig. 7), except subtraction of the dark current of the CMOS cameras (MUSE) or PMTs (NLM). For all other imaging results (Figs 2–6), the minimum value of each MUSE frame was also subtracted to suppress the background (Supplementary Fig. S2). Histogram brightness normalization was used to equalize the brightness of each fluorescent channels such that 0.01% of pixels were overexposed and lens vignette correction was used to facilitate seamless mosaicking. Virtual H&E rendering was performed using a virtual transmission microscopy (VTM) algorithm^18^. VTM is a physically realistic light absorption model that enables high quality rendering to resemble absorptive stains such as H&E by using fluorescent data and simulating the transmission of white light through virtual slides. Wide area mosaics were generated by stitching rendered virtual H&E frames using Image Composite Editor (Microsoft Research), enabling comparisons to conventional H&E histology slides.

### Data availability

Links to full resolution data of all mosaicked images presented are provided in the figure captions. Raw data generated during and/or analyzed during the current study and further details of the system design are available from the authors upon request.

## Electronic supplementary material

Supplementary text
