# Supplementary material for: Rapid histopathological imaging of skin and breast cancer surgical specimens using immersion microscopy with ultraviolet surface excitation
Source: Sci Rep. 2018 Mar 14;8:4476. doi: 10.1038/s41598-018-22264-2 (PMC5852098; doi:10.1038/s41598-018-22264-2)
Supplement: Supplementary file 1 — Supplementary text [file 41598_2018_22264_MOESM1_ESM.pdf]

SUPPLEMENTARY

TITLE

Rapid histopathological imaging of skin and breast cancer surgical specimens using immersion microscopy with ultraviolet surface excitation

AUTHORS

Tadayuki Yoshitake,[1] Michael G. Giacomelli,[1] Liza M. Quintana,[2] Hilde Vardeh,[2] Lucas C. Cahill,[1,3] Beverly E. Faulkner-Jones,[2] James L. Connolly,[2] Daihung Do,[4] and James G. Fujimoto[1,*]

AFFILIATIONS

[1]Massachusetts Institute of Technology, Department of Electrical Engineering and Computer Science and Research Laboratory of Electronics, 32 Vassar Street, Cambridge, MA 02139, USA
[2]Harvard Medical School, Department of Pathology, Beth Israel Deaconess Medical Center, 330 Brookline Avenue, Boston, MA 02215, USA
[3]Harvard-MIT Division of Health Sciences and Technology, 77 Massachusetts Ave, Cambridge, MA 02139, USA
[4]Harvard Medical School, Department of Dermatology, Beth Israel Deaconess Medical Center, 330 Brookline Avenue, Boston, MA 02215, USA
*Corresponding author: jgfuji@mit.edu

Propidium iodide staining with ethanol improves uptake in fresh specimens

PI is a widely used nuclear stain that is advantageous for MUSE imaging because of its high specificity for nuclei and long wavelength emission. We have previously demonstrated PI can rapidly stain fresh, unfixed tissue[4], although it is commonly used to preferentially stain cells with compromised cell membranes at lower concentrations. We have observed that although many cell nuclei in fresh, unfixed tissue are strongly stained with PI, dense ducts in freshly excised breast tissues sometimes fail to strongly stain, potentially causing in inconsistent visualization. To investigate this effect, fresh, unfixed human tissue (breast and skin) was stained using acridine orange (AO, a known live cell stain, but with lower specificity for nuclei than PI) at 40 µg/ml concentration and PI at 40 µg/ml concentration for 2 minutes. Water (Fig. S1a-c) or 70% ethanol (Fig. S1d-f) were used as a solvent. NLM imaging near the tissue surface down to 20 µm (the limit of MUSE excitation) showed that staining with PI dissolved in water had limited uptake in dense features such as TDLUs. However, PI dissolved in 70% ethanol was comparable to AO staining for ductal lobular units (Fig. S1d-f) when used in fresh, unfixed tissue. A 70% ethanol solvent was chosen because it is well known to disrupt cell membranes and PI is known to be more permeable in disrupted cell membranes. Repeating the experiment with fresh, unfixed skin (Fig.S1g-l) also showed that PI dissolved in 70% ethanol had improved uptake in fresh skin specimens, although the effect is less evident because features such as the epidermis are well visualized even with the reduced uptake associated with PI dissolved in water. In all cases, PI was more specific for cell nuclei than AO. These results demonstrate that special care must be taken with developing staining protocols using tissue specimens so that they can be translated to freshly excised, living tissue which would ultimately be encountered in intraoperative applications.

Comparison of MUSE images with and without fluorescent background subtraction

MUSE images have a high fluorescent background, but because it is relatively uniform, it can be estimated and subtracted, either on a per-frame basis (in real-time imaging) or on an overall basis (in mosaic imaging). In this study, we used real-time estimation by subtracting the minimum value in each channel of each acquired frame. After the subtraction, a scale factor is multiplied such that the maximum value of each channel in each frame remained unchanged (Fig. S2).

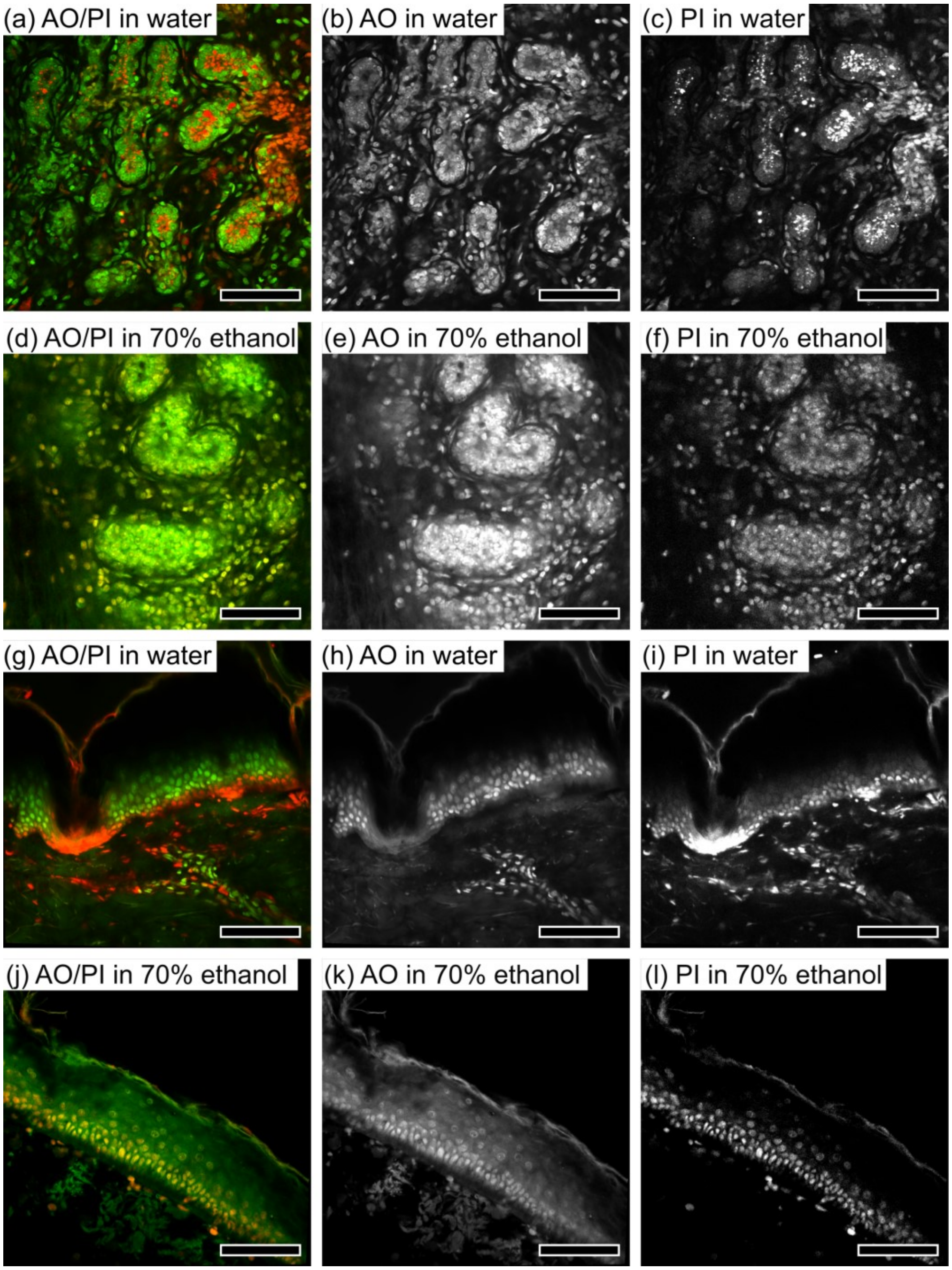

Fig. S1. AO/PI cell staining. Fresh, unfixed breast tissue stained with (a) AO/PI in water solution, (b) AO channel and (c) PI channel imaged using NLM at 20 μm below the tissue surface. Many cells that are visible under AO staining are not stained with PI. Fresh, unfixed breast tissue stained with (d) AO/PI in 70% ethanol solution, (e) AO channel, (f) PI channel imaged using NLM at 20 μm below the surface. The ethanol solvent improves the uptake of PI and nearly all cells are now visible under both AO and PI. Fresh, unfixed skin tissue stained with (g) AO/PI in water solution, (h) AO channel and (i) PI channel imaged using NLM at 20 μm below the tissue surface. Similar to the breast specimen, there are cells which are visible under AO staining which are incompletely stained by PI, although correspondence is higher. Fresh, unfixed skin tissue stained with (j) AO/PI in 70% ethanol solution, (k) AO channel, (l) PI channel imaged using NLM at 20 μm from surface. The uptake of PI in skin is improved by the ethanol solvent. Scale bar, 100 μm.

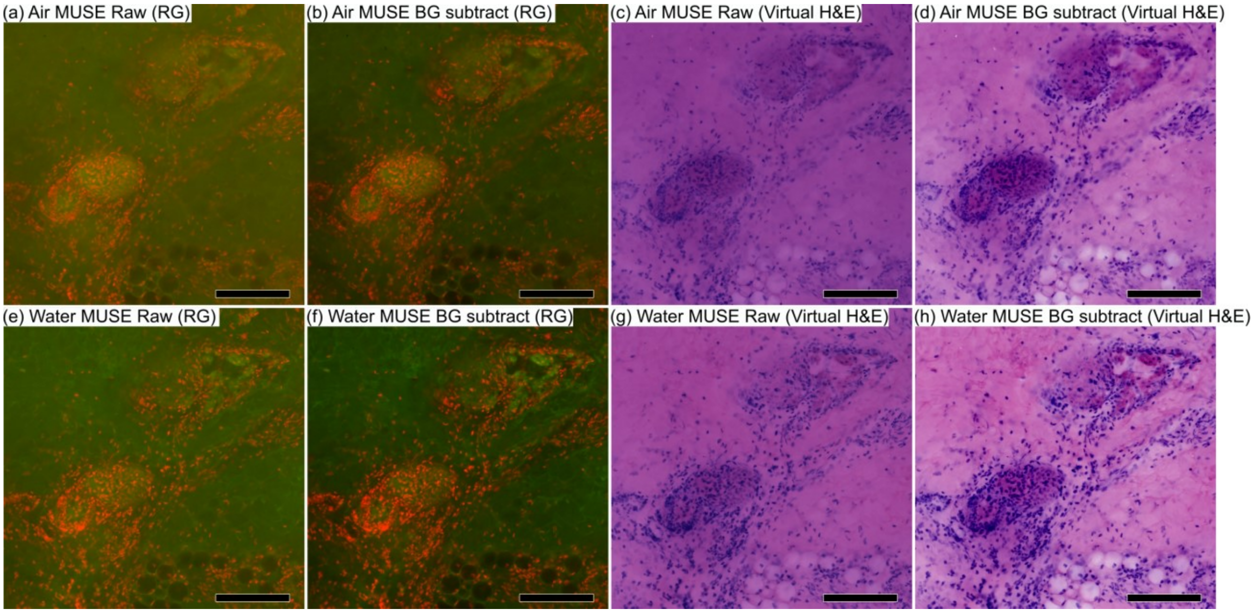

Fig. S2. Comparison of MUSE virtual H&E images with and without background subtraction. (Fresh, unfixed normal skin) MUSE images of normal human skin specimen acquired with air immersion MUSE; (a) raw image (RG), (b) uniform background subtracted image (RG), (c) raw image rendered as virtual H&E, and (d) background subtraction & virtual H&E rendered image. Images acquired with water immersion MUSE; (e) raw image (RG), (f) background subtracted image (RG), (g) raw image & virtual H&E rendering, and (h) background subtraction & virtual H&E rendered image. Background subtraction has a pronounced effect on air immersion MUSE images due to the large background signal. Scale bar, 100 µm.
